# Cell Surface Receptors for Signal Transduction and Ligand Transport: A Design Principles Study

**DOI:** 10.1371/journal.pcbi.0030101

**Published:** 2007-06-01

**Authors:** Harish Shankaran, Haluk Resat, H. Steven Wiley

**Affiliations:** Systems Biology Program, Pacific Northwest National Laboratory, Richland, Washington, United States of America; Harvard University, United States of America

## Abstract

Receptors constitute the interface of cells to their external environment. These molecules bind specific ligands involved in multiple processes, such as signal transduction and nutrient transport. Although a variety of cell surface receptors undergo endocytosis, the systems-level design principles that govern the evolution of receptor trafficking dynamics are far from fully understood. We have constructed a generalized mathematical model of receptor–ligand binding and internalization to understand how receptor internalization dynamics encodes receptor function and regulation. A given signaling or transport receptor system represents a particular implementation of this module with a specific set of kinetic parameters. Parametric analysis of the response of receptor systems to ligand inputs reveals that receptor systems can be characterized as being: i) avidity-controlled where the response control depends primarily on the extracellular ligand capture efficiency, ii) consumption-controlled where the ability to internalize surface-bound ligand is the primary control parameter, and iii) dual-sensitivity where both the avidity and consumption parameters are important. We show that the transferrin and low-density lipoprotein receptors are avidity-controlled, the vitellogenin receptor is consumption-controlled, and the epidermal growth factor receptor is a dual-sensitivity receptor. Significantly, we show that ligand-induced endocytosis is a mechanism to enhance the accuracy of signaling receptors rather than merely serving to attenuate signaling. Our analysis reveals that the location of a receptor system in the avidity-consumption parameter space can be used to understand both its function and its regulation.

## Author Summary

Cells interact with their environment using molecules on their surface known as receptors. Receptors bind specific companion molecules known as ligands, which either carry information about the outside environment or are critical cell nutrients. Signaling receptors bind the former ligand type and convert information about the outside environment to a cell response such as migration or growth. Transport receptors bind the latter class of ligand and deliver them to the cell interior. A variety of receptors are internalized into the cell through a process known as endocytosis. Receptors display a wide range of endocytosis patterns, but the functional motivation behind the observed differences is not well understood. We have constructed a generalized model to understand how receptor endocytosis and other receptor–ligand properties affect the function of receptor systems. We find that the efficiency and robustness of receptor systems are encoded by two fundamental parameters: i) the *avidity* which quantifies the ability of a receptor system to capture ligand, and ii) the *consumption* which quantifies the ability to internalize bound ligand. By examining a number of receptor systems, we demonstrate that the internalization dynamics of receptor systems can be explained by examining its effect on the avidity and consumption parameters.

## Introduction

There is considerable evidence to suggest that biomolecular networks are optimally evolved so that they are efficient and function in a robust fashion [1–5]. An additional feature of human engineered as well as biological systems is modularity [6–8]. In the context of cell signaling, a functional module represents a relatively self-contained subnetwork with a specific topology that is designed to perform a specific function. However, it is important to note that the internal system parameters of these modules are not fixed. For instance, the kinetic parameters of a given cell-signaling module can be tuned to achieve different levels of efficiency and robustness. This property would allow a module to be reused in a wide variety of cellular contexts with the characteristics of the module being tailored to suit the task at hand. From a reverse-engineering standpoint, understanding the design principles of a specific module would further the understanding of the entire set of networks that the module is a part of. Hence, adopting a module-based rather than a molecule-based approach should greatly facilitate the task of obtaining a systems-level understanding of cell function [6].

In this manuscript we employ a module-based approach to characterize the design principles of cell surface receptor systems. In so doing, we illustrate a novel strategy that we believe can be fruitfully applied to a wide range of systems biology problems. Our general approach can be outlined as follows. First, we create a generalized mathematical model for the upstream biomolecular network employed by a diverse set of cell surface receptors. Here, the biomolecular network is the module, and a given receptor system represents a particular implementation of the module with a characteristic set of kinetic parameters. We establish quantitative metrics that can be used to assess network function and robustness. We analyze the model in the context of these metrics to establish the critical “control parameters,” and to characterize the behavior of the module in various regions of the control parameter space. Finally, we map experimentally determined kinetic parameters for different receptor systems onto the control parameter space. The location of the various receptor systems in control parameter space prescribes their specific function and regulation. We validate our analysis methodology by comparing model predictions with experimental observations on the function and regulation of the chosen receptor systems. We find that our results provide significant insights regarding differences in the dynamic properties of the investigated receptor systems.

Receptors constitute the interface of cells to their external environment. These molecules bind specific ligands involved in multiple processes, such as signal transduction and nutrient transport. It has long been appreciated that binding specificity is a central feature of receptor function, and numerous studies have explored the structural elements involved in this aspect [9–11]. An equally important function of many classes of receptors is their ability to transport bound molecules into cells by receptor-mediated endocytosis. In the case of transport receptors, such as those that bind transferrin and low-density lipoprotein, this process serves the obvious role of transporting molecules to their site of utilization. In the case of signaling receptors, such as those that bind growth factors and cytokines, endocytosis putatively consumes the information-containing ligands, allowing cells to respond to a new stimulus. Although the molecular mechanisms of endocytosis have been explored for many years [12], the systems-level functional constraints that drive the evolution of receptor dynamics have not been addressed.

Receptor behavior is governed by the physical aspects of ligands as well by as the kinetic properties of the receptor. As an example of a physical constraint, ligands and receptors must evolve together as cohorts because both are necessary for system function. The functional constraints of this co-evolution include the specificity and affinity of the ligand–receptor binding reaction [10]. Dynamic aspects of receptor behavior are also functionally important and thus subjected to evolutionary pressure. For example, following endocytosis, transferrin releases its iron in the acidic environment of the endosome and recycles back to the cell surface while still bound to its receptor [13]. In contrast, low-density lipoprotein dissociates from its receptor and is degraded in lysosomes while its receptor recycles back to the cell surface [14]. These behaviors make sense in terms of metabolic efficiency because of the obvious advantage of reusing transport molecules as many times as possible.

It is relatively easy to envision the system constraints that drive the evolution of transport receptors. However, the factors that constrain signaling receptors are far less clear because biology lacks a coherent theory on how biological information is encoded. In the case of receptor systems that detect physical aspects of the environment, such as chemotactic gradients, the information content is direct and system properties necessary to decode it are conceptually straightforward (e.g., adaptive responses). In multicellular organisms, however, much of the information transmitted between cells is encoded in the form of growth factors and cytokines. It is known that information can be encoded in ligand structures and in the rate, duration, and intensity of ligand production, but the mechanisms by which receptor systems decode this information is poorly understood. How the need to decode dynamic information influences the kinetic properties of growth factor receptors has not previously been investigated.

It has been difficult to explore the functional significance of specific aspects of receptor behavior using traditional experimental systems. Mutational approaches have been used to alter receptor binding and internalization characteristics [15,16], but changing receptor structure can cause numerous confounding effects, such as alterations in its ability to interact with substrates or other binding proteins. It is also difficult to investigate receptor behavior under dynamic conditions that mimic a reasonable physiological context. For example, ligands are generally added experimentally as a bolus (one-time instantaneous ligand addition), but this sort of quantal change in ligand concentration is rarely encountered in situ. Cells that artificially produce ligands in a regulated fashion have been constructed to obviate some of these problems, but their complexity restricts them to the exploration of relatively simple questions [17]. Fortunately, many of the quantitative parameters that govern the dynamics of ligand–receptor systems have been measured and characterized. These parameters provide a foundation for constructing mathematical models of ligand–receptor interactions that can be used to explore the functional significance of different aspects of receptor dynamics.

In this study we employed mathematical modeling to compare the characteristics of four well-defined yet distinct receptor systems: epidermal growth factor, transferrin, low-density lipoprotein, and vitellogenin. These receptors mediate three distinct physiological functions: cell signaling, nutrient import, and protein transport. This study was undertaken to explore the general design principles underlying receptor dynamics. Specifically, we were interested in understanding why receptors show a wide diversity in their rates of endocytosis and trafficking. The four systems considered here are highly regulated receptor systems and they have been subjected to extensive kinetic characterization. Thus, sufficient quantitative information is available to support their comparative analysis.

The epidermal growth factor receptor (EGFR) is an important receptor in the context of development and tumorigenesis [18–20]. The binding of its ligand activates downstream signaling pathways such as the MAPK and PI3K/Akt pathways [19]. One of the most intriguing properties of the EGFR is enhanced endocytosis following occupancy. Although the EGFR is probably the most extensively characterized receptor that displays this property, it occurs in many other receptors, too [21–23]. The activation of the EGFR by ligand binding causes a nearly 10-fold increase in the rate of receptor internalization and a rapid loss of receptors from the cell surface [24]. This accelerated endocytosis has been postulated to be the primary mechanism responsible for receptor “downregulation” in which the rate of receptor degradation is also increased following their activation [24]. However, there are many examples of activated receptors that are degraded at an accelerated rate despite an invariant rate of endocytosis [25,26]. Recent studies have shown that the mechanistic basis of accelerated endocytosis and degradation are quite distinct and mediated by different receptor domains and intracellular processes [27,28]. Biochemical and kinetic studies have also shown that a complex regulatory system is involved in accelerated endocytosis, suggesting that it plays a crucial role in receptor regulation [29] .

The transferrin receptor (TfR) plays an important role in the cellular uptake of iron from the extracellular space [30]. Long-term regulation of the level of TfR expression is controlled by both iron-mediated receptor transcription [31] and the regulated stability of the TfR mRNA [32,33]. Levels of surface TfR can be modulated acutely by the actions of growth factors, which cause a redistribution of intracellular receptors to the plasma membrane [34,35]. Like most other receptors involved in nutrient transport, the TfR is internalized at a constant rate regardless of its occupancy state [36]. The internalized receptor is recycled back to the plasma membrane once iron dissociates from transferrin in the low pH environment inside the cell.

The low-density lipoprotein receptor (LDLR) mediates the supply of cholesterol to the cell interior by virtue of its ability to bind and internalize low-density lipoprotein [37]. Following internalization, the ligand is degraded in lysosomal compartments, and the cholesterol is liberated for cellular use [37]. Ligand-free receptors are then recycled back to the cell surface. The LDLR is subjected to rapid turnover even in the absence of ligand with free receptors being constantly internalized and recycled. LDLR internalization rates are not significantly affected following ligand binding [38]. The cell surface expression level of LDLR is transcriptionally regulated by hormones and growth factors such as growth hormone [39,40], phorbol 12-myristate 13-acetate (PMA) [41], and hepatocyte growth factor [42].

The vitellogenin receptor (VtgR) is a transport receptor that plays an important role during oogenesis in many oviparous species, including birds, frogs, and fish [43,44]. During the growth phase of *Xenopus laevis,* 90% of total oocyte protein is derived from the specific uptake of the protein vitellogenin from the maternal bloodstream [45]. Internalized vitellogenin is proteolytically converted to the yolk proteins prior to their storage as crystalline inclusions, termed yolk platelets [46]. Similar to the TfR and the LDLR, the net rate of VtgR internalization is independent of ligand binding [47]. However, the specific endocytic rate of the VtgR is under hormonal regulation [48].

Using a simple mathematical model for a canonical receptor–ligand binding and trafficking module, we investigated the relationships between the kinetic characteristics and the specific physiological functions of the EGFR, TfR, LDLR, and VtgR systems. Our analysis of the mathematical model reveals that module efficiency and robustness are encoded by two dimensionless parameters: i) a specific avidity parameter *γ* representing the efficiency of ligand capture from the extracellular space, and ii) a partition coefficient *β,* which is the probability that a captured ligand molecule will be internalized before it dissociates from the receptor. We found that the module exhibits different properties depending upon its parameter values and can be characterized as being: i) avidity-controlled where the response depends primarily on the avidity, ii) consumption-controlled where the consumption is the primary control parameter, and iii) dual-sensitivity where both *β* and *γ* are important. Significantly, we found that the location of different receptor types in the *β–γ* parameter space correlates with their specific function and regulation. However, all receptor types appeared to be suboptimum with respect to system robustness, apparently because of the necessity of cells to be able to control receptor activity. Our module-based mathematical analysis suggests that a set of general design principles can be used to understand receptor dynamics and the opposing needs for robustness and regulation.

## Results

### Model Description

We have previously described several detailed kinetic models of the binding, internalization, and degradation of polypeptide ligands [49–52]. However, to facilitate the comparison of multiple receptor systems, we revert to a canonical model that is simple yet encompasses the salient features of the investigated systems (Figure 1). We note that the spatio–temporal distribution of activated EGFR obtained using the current model is similar to results (unpublished data) obtained using extended models that we have previously employed [49,50].

**Figure 1:**
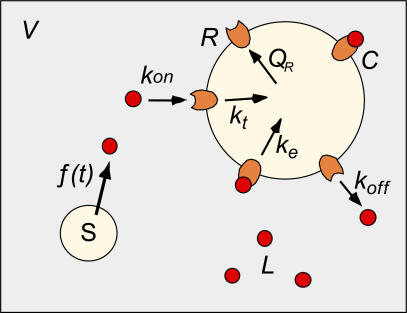
Schematic Representation of the Receptor–Ligand Binding and Internalization Model The reactant species in the model are the ligand *L*, free receptor *R*, and receptor–ligand complex *C*. Ligand is produced at a rate *f*(*t*) by a source *S* and enters the extracellular space with volume *V*. Ligand reversibly binds free receptors with a forward rate *k_on_* and a reverse rate *k_off_* to yield receptor–ligand complexes. Free receptors are synthesized by the cell at a rate *Q_R_*. Free receptors and receptor–ligand complexes are internalized with the rates *k_t_* and *k_e_*, respectively. The input to the above system is the external ligand stimulus *f*(*t*), and the output is the concentration of surface complexes *C*.

In our model, ligand is produced at a specific rate *f*(*t*) by a source S and enters a well-mixed volume *V*, building up to a concentration *L*. The ligand reversibly binds free surface receptors *R* with forward rate *k_on_* and reverse rate *k_off_* to form a receptor–ligand complex *C*. The empty and occupied receptors are internalized with characteristic rate constants *k_t_* and *k_e_*, respectively. The act of internalization consumes the receptors and ligands. For the purpose of our general analysis, the input parameter is the production rate of ligand over time *f*(*t*), and the output is the level of occupied receptors at the cell surface *C*.

The rates of change for the various species in our model can be written as:

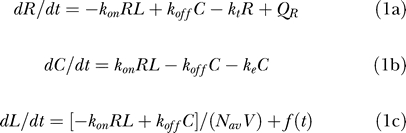
where *N_av_* is Avagadro's number and *V* is the volume of extracellular medium per cell. In Equation 1, *R* and *C* are expressed in molecules, *L* is a concentration in nM unit, *f*(*t*) has units of nM/min, and the rate constants have the units listed in Table 1. A typical cell culture experiment would have 10 ml of media and a cell count of 2.5 × 10^7^ cells. Thus *V* = 4 × 10^−10^ liters/cell. The factor in the denominator of the first term of Equation 1c ensures conversion of the ligand consumption term from molecules to a molar concentration. When there is no extracellular ligand present *L*(*t*) *= C*(*t*) *=* 0, we obtain the value *R_T_* = *Q_R_*/*k_t_* for the steady state surface receptor abundance. This expression states that the total number of surface receptors prior to ligand addition reflects the balance between receptor synthesis and internalization terms. In all, we have six independent parameters in our model: *k_on_*, *k_off_*, *k_e_*, *k_t_*, *V*, and *R_T_*. Further details of our model and the solution methodology for the reaction system described by Equation 1 are provided in the Methods section.

**Table 1:**
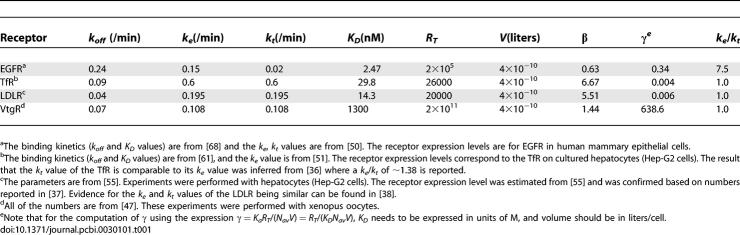
Receptor–Ligand Binding and Internalization Parameters for Receptor Systems

As typically done in kinetic studies, complex aspects of the receptor dynamics are subsumed by “lumped” parameters in the model. For example, the parameter *k_on_* contains factors such as diffusivity of the ligand and steric aspects of the ligand–receptor binding pocket. The rate of receptor production, *Q_R_*, combines the parameters for net receptor synthesis and receptor recycling. Using *C* as a system output parameter is reasonable for a variety of reasons. First, in the case of the EGFR and other signaling receptors, the biological response has been shown to be proportional to the number of receptor–ligand complexes at the cell surface [53,54]. Further, in most cases, there are “spare receptors” relative to the number required to produce a maximal biological response, suggesting that the number of occupied receptors at the cell surface will be the controlling factor to downstream events [55]. Most important, this model captures the parameters common to many different receptor systems, thus allowing us to understand the relationship between these parameters and receptor function.

### Comparative Dynamics of Receptor Systems: Relationships between Kinetic Parameters and Receptor Dynamics

Our objective in this study was to understand how the kinetic properties of receptor systems influence receptor function and to use this knowledge to uncover the general design principles of receptor systems. As seen in Table 1, the four receptor systems we consider span a wide range of parameter values. In particular, the receptor systems display a wide variation in affinity *K_a_* (the reciprocal of the dissociation constant *K_D_* = *k_off_*/*k_on_* = 1*/K_a_*) and receptor expression *R_T_*, while the other parameters tend to vary in a narrower range. We hypothesized that examining the dynamics of these receptor systems should indicate the relationships between system parameters and receptor function. Further, quantifying the sensitivities of each receptor system to variations in individual system parameters should reveal differences in receptor regulation patterns. Thus, we employed our mathematical model to simulate the response of each of our specific receptor systems to ligand impulses and step changes in ligand entry. First, we examined the sensitivity of receptor dynamics to two specific parameters, the endocytosis rate of occupied receptors *k_e_*, and the extracellular volume *V.* The reason we choose these specific parameters will be readily apparent when we examine the dimensionless version of the governing equations for our model. Unless specified otherwise, results presented in this section were obtained by solving the governing equations (Equation 1) using the kinetic parameters listed in Table 1.

#### Endocytic downregulation improves the information processing accuracy of the EGFR.

A distinguishing kinetic feature among many growth factor receptor systems is occupancy-induced receptor loss, also known as endocytic downregulation. Whereas the signaling receptor EGFR displays endocytic downregulation [24], the internalization rates of TfR, LDLR, and VtgR are not significantly altered following ligand binding [36,38]. Thus, it is likely that endocytic downregulation confers some functional advantage to signaling receptors. To examine this possibility, we studied the effect of altering the magnitude of endocytic downregulation on the ability of the EGFR system to decode time-varying ligand inputs (Figure 2). For these simulations, the endocytic downregulation ratio *D* was quantified as the ratio of the internalization rates of occupied versus unoccupied receptors (*D = k_e_*/*k_t_*). This was varied in the range 1–20 by changing the value of *k_e_* while keeping *k_t,_* and all the other parameter values constant. We note that the experimentally measured endocytosis rate of EGFR is within this range. Figure 2A presents the response of the EGFR system to an input ligand pulse with a total magnitude of 0.01 *K_D_*. All subsequent impulse response calculations in the manuscript are also performed at this ligand dose. This corresponds to the instantaneous addition of a ligand dose of concentration 0.01 *K_D_* nM to the extracellular volume at time *t* = 0. Our choice of a low ligand concentration for the impulse response calculations was motivated both by biological and theoretical reasons. First, such small perturbations are likely to be more representative of physiological ligand dosages. In particular, for signaling receptors such as the EGFR, we and others have shown that the ligand release rates are such that the concentration of extracellular ligand would be low [17,56]. Second, in the case of the EGFR, the occupancy of only a small number of receptors is sufficient to trigger a biological response. Furthermore, the chosen ligand dosage also enables us to assess the behavior of the model in its linear regime. This allows us to employ an analytical solution that greatly facilitates assessment of the properties of the model.

**Figure 2:**
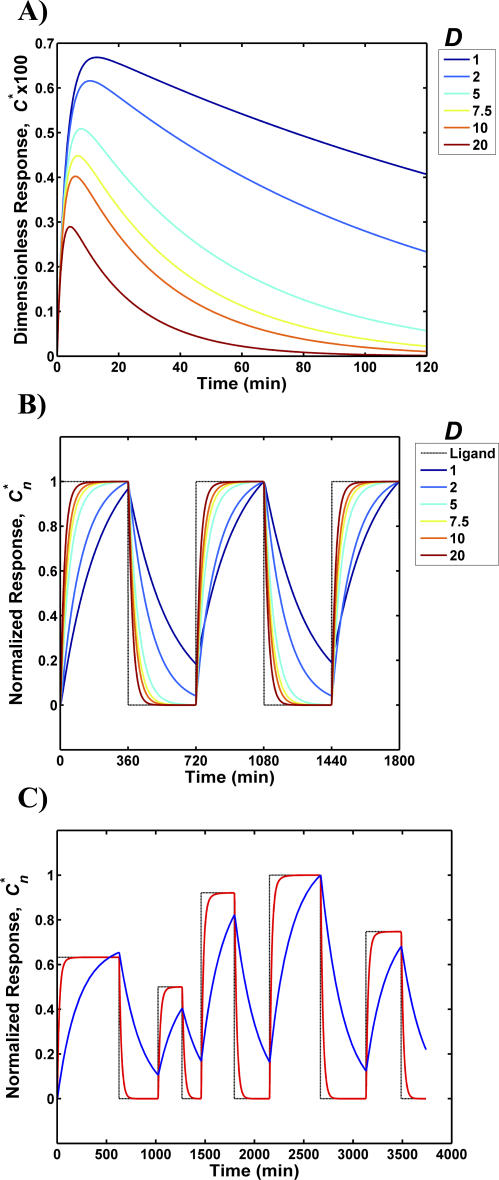
Effect of Downregulation on the Accuracy of EGFR Information Processing The dimensionless response C^*^ and the normalized response C_n_^*^ of the EGFR system were computed as a function time for various downregulation magnitudes, *D*. (A) Impulse response of the EGFR system for various *D* values. (B) Normalized response to step changes in the ligand input rate to the system. The input—denoted by a block dotted line—consists of a 360-min stimulus phase where ligand enters the system at a constant rate followed by a 360-min recovery phase where ligand input is set to zero. (C) Normalized response to a non-uniform step input. The mean value of the ligand entry rate was set such that at steady state sustained ligand release at this rate would leave 95% of the receptors in the unbound form. The mean values of the durations of the on-and-off phases of the step input were set to equal 400 min. The actual ligand entry rate and duration for each pulse was sampled from a normal distribution with a standard deviation of 50%. The response of the EGFR system is plotted for *D* = 1 (blue line) and *D* = 20 (red line). The normalized input waveform is shown as a black dotted line.

As seen in Figure 2A, the EGFR impulse response was characterized by a rise in the number of complexes to a peak value followed by a subsequent decay to zero. The rise was the consequence of the formation of new receptor–ligand complexes, while the decay reflected the internalization of complexes and the depletion of extracellular ligand. Increasing the magnitude of endocytic downregulation increased the responsiveness of the system. Both the time taken to reach the peak value and time to decay were decreased when *D* was increased. However, increasing *D* resulted in smaller response amplitudes (Figure 2A) because of the net loss of surface receptors that occurred when ligand-induced endocytosis was higher. These impulse response results have implications for the system's ability to decode ligand pulse trains consisting of successive spikes because a faster impulse response time would allow the system to accurately transduce higher-frequency pulse trains. The qualitative behavior of the impulse response does not depend on the ligand concentration. Specifically, if these numerical experiments are repeated using higher ligand dosages (a condition that better reflects the typical in vitro cell culture experiments), conclusions that are based on the trends in the response time will not change. The most obvious changes will be in the amplitude of the response, which is to be expected.

We further examined the effect of endocytic downregulation on EGFR signal processing by considering the response to a periodic square wave ligand input. In Figure 2B, the normalized response C_n_^*^ is plotted as a function of time for various *D* values. For these simulations, the step input corresponds to a 360-min phase where ligand was added to the system at a constant rate followed by a 360-min relaxation phase where the ligand entry rate was zero. The ligand entry rate in the stimulus phase was set such that at steady state, 95% of the total receptors would remain as free unoccupied receptors if the ligand addition was sustained at that level. Thus, these square-wave ligand inputs also constitute relatively small perturbations to the system. As seen in Figure 2B for the case where there is no endocytic downregulation (*D* = 1), the system output has a sawtooth appearance and bears little resemblance to the square wave input. Increasing *D* leads to outputs that more closely resemble the ligand input waveform.

To better understand the implications of different values of *D* on the ability of cells to process ligand information, we simulated step inputs with non-uniform variations in the ligand addition and relaxation durations (Figure 2C). A mean duration of 400 min each was chosen for the ligand addition and the relaxation phases, but the actual duration for each random pulse was sampled from a Gaussian distribution with a 50% standard deviation. When *D* = 20, despite the irregular nature of the input pulse train, the response was a faithful reproduction of the information contained in the input in terms of both time and magnitude (red line). On the other hand, when there was no endocytic downregulation (*D* = 1), the shape and magnitude of the system output was poorly correlated to the input pulse train (blue line). Clearly, increased endocytic downregulation improves the information processing accuracy of the EGFR system.

#### Comparison of the effect of occupancy-induced internalization on receptor function.

We explored whether the sensitivity displayed by the EGFR to variations in endocytic downregulation was a general feature of all receptors, or whether it was dependent on the characteristic parameters of the EGFR. To address this issue, we evaluated the sensitivity of the receptor dynamics to changes in the *k_e_*/*k_t_* ratio for each of the four receptor systems listed in Table 1 (Figure 3). Because most of the receptor systems were transport receptors, the amount of internalized ligand was used to assess their performance. Specifically, the response to a ligand impulse of magnitude 0.01*K_D_* was simulated for different *k_e_* values, and the variation in internalized ligand was plotted as a function of time. All the other parameters were those specified in Table 1. The number of internalized ligand molecules was taken to be equal to the number of internalized receptor–ligand complexes, which was determined by integrating the product *k_e_C*(*t*). Hence, we express the cumulative internalized ligand as a percentage of the total amount of ligand initially added to the system. Note that the panels in Figure 3 employ different time scales to facilitate an easier visual comparison. Time ranges for each case were chosen such that the fastest curve (red curve) reaches a magnitude of 80% when 20% of the time has elapsed. As seen in Figure 3, the receptor systems display a wide variation in minimum response times with the speed increasing on the order VtgR > EGFR > TfR > LDLR.

**Figure 3:**
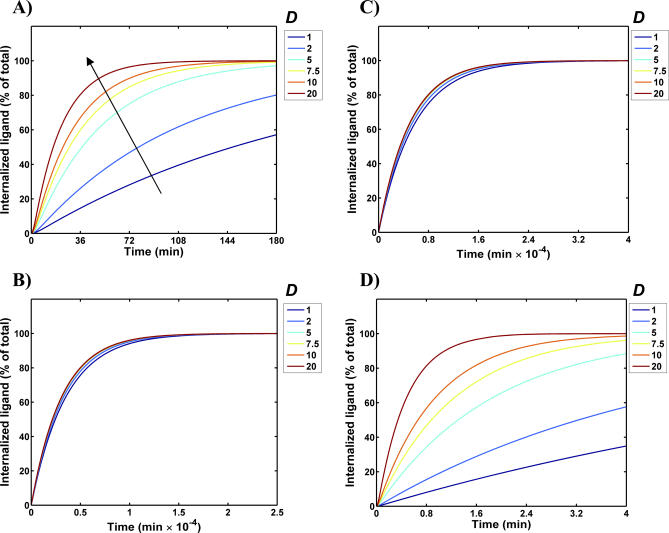
Effect of Altering *k_e_* on the Impulse Response of the Receptor Systems The increase in internalized ligand following a ligand impulse was computed as a function time for the various receptor systems. Internalized ligand concentrations are presented as a fraction of the total ligand dose added. Results are presented for EGFR (A), TfR (B), LDLR (C), and VtgR (D). For each receptor system, *k_e_* was varied to obtain the various *D* values (*k_e_*/*k_t_* ratios) indicated, while all other parameters were set to equal the values listed in Table 1. The arrow in (A) denotes the direction of increasing *k_e_*. Note that the subplots have different time scales. To clarify the axis labeling, the *x*-axis ranges from 0 to 25,000 min and 0 to 40,000 min in (B) and (C), respectively.

We observed that although the response speeds of the EGFR (Figure 3A) and the VtgR (Figure 3D) were sensitive to variations in the value of *k_e_* relative to *k_t_*, the temporal response patterns of the TfR (Figure 3B) and LDLR (Figure 3C) were mostly unaffected by variations in *k_e_*. Thus, endocytic downregulation is an effective strategy for increasing response times only in the context of a specific set of receptor parameters. This indicates that analyzing response sensitivity to a single kinetic parameter is insufficient for understanding the functional characteristics of a receptor system.

#### Sensitivity of receptor function to system volume.

The extracellular volume is a unique model parameter in that it is independent of receptor and ligand properties. Receptor–ligand systems presumably evolve in a specific physical context and are likely to be optimized for a specific extracellular volume or range of volumes. A receptor system that is required to function in a broad range of extracellular volumes needs to be relatively robust to volume changes. On the other hand, a receptor system that only encounters a narrow range of volumes in vivo would not be impaired by sensitivity to volume changes. To understand the design principles of receptor systems, it is thus instructive to examine the effect of volume changes on the receptor response. We simulated the effect of changing the volume in a broad range between 4 × 10^−13^ liters/cell to 4 × 10^−10^ liters/cell on the impulse response of our four model receptors (Figure 4). The smallest extracellular volume used in the simulations corresponded to approximately one cell volume per cell. This is a reasonable value for interstitial tissue volume and represents the situation where a growth factor is released and consumed locally (i.e., an autocrine or paracrine growth factor). The upper volume limit approximates tissue culture systems or the circulating volume in the body. We note that ligand secretion and diffusion processes have been explicitly modeled by others using partial differential equation based formalisms and stochastic simulations [57,58]. By comparing results from our simplified model with these spatial simulations, it should be possible to determine the apparent extracellular volume to use in our model for various ligand consumption and intercellular communication scenarios.

**Figure 4:**
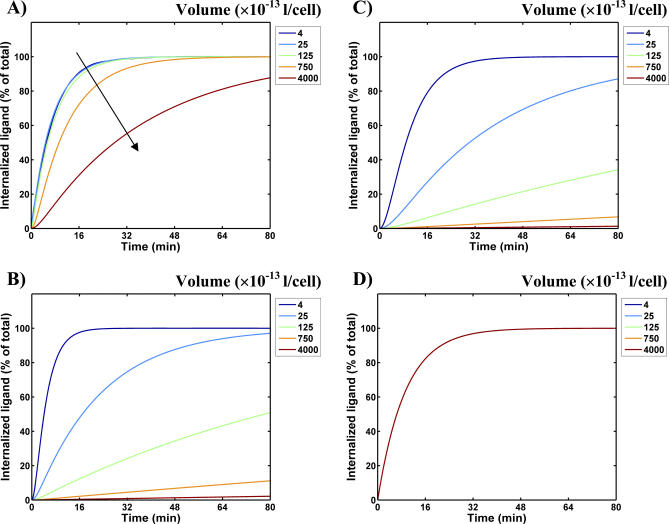
Sensitivity of Receptor Function to Extracellular Volume The increase in internalized ligand following a ligand impulse is plotted as a function of time for various extracellular volumes for EGFR (A), TfR (B), LDLR (C), and VtgR (D). The volume *V* was varied as indicated in Figure 4, while all other parameters were set equal to the values listed in Table 1. The arrow in (A) denotes the direction of increasing volume.

As seen in Figure 4A, the EGFR is relatively insensitive to volume changes in a two-orders-of-magnitude range from 4 × 10^−13^ liters/cell to 4 × 10^−11^ liters/cell. This would suggest that the EGFR system is capable of functioning robustly over a reasonably broad range of volumes. In contrast, the TfR (Figure 4B) and the LDLR (Figure 4C) systems display a much greater sensitivity to volume changes. Surprisingly, the response of the VtgR system (Figure 4D) appears completely refractory to changes in extracellular volume. The reason for the disparate response of the different receptor systems to changes in the extracellular volume is not apparent from a simple inspection of their parameters. This suggests that the optimization of receptor behavior involves a non-intuitive interaction of multiple system parameters.

### Receptor Behavior as a Function of Two Dimensionless Parameters

As the above reported results indicate, examining the contributions of individual parameters to receptor dynamics may not be adequate to improve our understanding of how the kinetic parameters encode the physiological function of receptor systems. To better understand the relationship between parameters and receptor function, we converted the model equations to a dimensionless form. The dimensionless governing equations for the system are as follows:

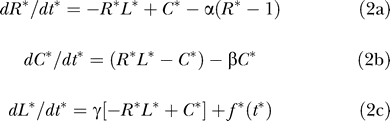
In Equation 2, R^*^ = R/R_T_ , C^*^ = C/R_T_ , L^*^ = L/K_D_ are the normalized species abundances, t^*^ = tk_off_ is the dimensionless time, f^*^(t^*^) is the dimensionless time-dependent ligand entry rate, and *K_D_* = *k_off_*/*k_on_* is the dissociation constant.

There are three apparent dimensionless parameters in the system that emerge naturally during the course of the conversion, viz. *α* = *k*_t_/*k_off_, β* = *k*_e_/*k_off_*, and *γ* = *K_a_R_T_*/(*N_av_V*) where *K_a_* = *k_on_*/*k_off_* is the receptor–ligand binding affinity. However, we are interested in how the system responds; that is, how the number of receptor–ligand complexes C^*^ evolves as a function of time for specified ligand inputs f^*^(t^*^). It can be shown that the response C^*^(t^*^) for any given input f^*^(t^*^) is a function only of two parameters viz. *β* and *γ*. Here *γ* is the specific avidity characterizing how efficiently a receptor system can capture extracellular ligand. The parameter *β* is the partition coefficient quantifying the probability that a captured ligand molecule will be internalized before it dissociates from the receptor. Hence, the partition coefficient can also be viewed as a consumption parameter because it quantifies how well a cell can internalize or consume surface-bound ligand molecules. With respect to our simulation results that were reported in the previous section, increasing the *k*_e_ value (Figure 3) corresponds to increasing the consumption parameter *β* of a receptor system. Decreasing the extracellular volume *V* (Figure 4) is equivalent to increasing the specific avidity *γ*.

As discussed above, the speed of the response of a receptor system to changes in ligand production appears to be a good way to assess system function for both signaling and transport receptors. For signaling receptors such as the EGFR, faster response times reflect better information processing accuracy. For transport receptors such as TfR, LDLR, and VtgR, the impulse response speed is directly related to the net ligand internalization rate, and hence faster responses reflect higher uptake efficiencies. Here, we computed the dimensionless relaxation time *τ* for the response to a ligand impulse of magnitude equal to 0.01*K_D_* and used this as a common metric to assess receptor performance. The relaxation time *τ* is the time taken for the impulse response (see Figure 2A for example) to decay to a value of 1/e of the peak value.

The *τ* value is inversely related to the response speed. A smaller relaxation time implies a faster response and hence indicates better receptor system performance. Dependence of *τ* on the dimensionless model parameters *β* and *γ* is examined in Figure 5. The *β* and *γ* values for the specific receptor systems considered here are shown overlaid on the panels in Figure 5. These values were computed using the information tabulated in Table 1. The dimensionless relaxation time *τ* varies over a wide range from 10^−2^ to 10^6^ when *β* and *γ* values are varied in their respective physiological ranges (Figure 5A). For small values of *β* and *γ*, the impulse response is characterized by large relaxation times (red region in Figure 5A). When *β* and *γ* are increased, the relaxation time decreases, with the smallest relaxation times being found in regions where both of the parameters are high (blue region). These findings can be explained based on the physical meanings of these parameters. The specific avidity *γ* is a measure of the efficiency of ligand capture from the extracellular space. The consumption parameter *β* represents the efficiency with which ligand captured by the cell surface receptors can be sequestered into intracellular compartments. Thus, the two parameters complement each other in determining the efficiency of ligand transport into the cell, and, therefore, an increase in either parameter leads to the shortening of the relaxation time.

**Figure 5:**
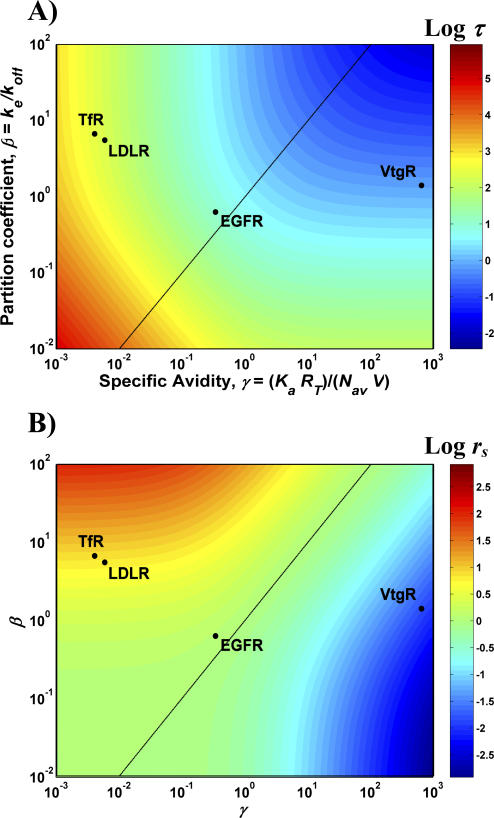
Relaxation Time and Its Sensitivity to Variations in System Parameters The relaxation time *τ* was computed for a range of specific avidity and partition coefficient values by solving the differential equations governing the system. (A) Dimensionless relaxation time *τ* is plotted as a function of *β* and *γ*. Blue regions correspond to small relaxation times and hence a rapid response, while red regions correspond to large relaxation times and a sluggish response. (B) The relative sensitivity of the relaxation time to changes in *β* and *γ, r_s_*. Blue regions indicate sensitivity primarily to *β* alone, and red regions indicate sensitivity to *γ* alone. The green region in the middle indicates approximately equal sensitivities to *β* and *γ*. The *β* and *γ* values corresponding to the TfR, LDLR, EGFR, and VtgR are marked in each of the panels. The main diagonal, *β = γ* is shown as a black line.

The locations of the various receptor systems in the *β–γ* parameter space specify their respective response speeds. The locations of the EGFR, TfR, LDLR, and VtgR in the *β–γ* parameter space are consistent with the impulse response speeds seen in Figures 3 and 4. Note that the dimensionless *τ* values in Figure 5A have to be divided by the respective *k_off_* values prior to comparison with the results plotted in Figures 3 and 4.

The relative sensitivity of the relaxation time to changes in *β* and *γ* provides us with specific information on the strategy that a receptor system operating at a given point in the parameter space could employ to improve its response. For this purpose we define a relative sensitivity *r_s_* parameter, which was computed as the ratio of the sensitivity of *τ* to *γ* to the sensitivity of *τ* to *β* as described in Methods. This quantity varies from 10^−3^ to 10^3^ in the parameter range studied (Figure 5B). Hence, depending upon the location in the parameter space, the relaxation time can be up to 1,000 times more sensitive to one of the parameters compared with the other. At high values of *β* (*β* > 10), the relaxation time is far more sensitive to the avidity *γ* than the consumption (red region). In this region of the parameter space, changes in the value of *γ* would be much more effective in decreasing response times. Similarly, for high *γ* values (*γ* > 10; blue region) the sensitivity to *β* is orders of magnitude greater than the sensitivity to *γ*. In this region of the parameter space, only changes in the value of *β* would be effective in optimizing response times. For the region where *r_s_* is close to 1 (near the diagonal, green region), modifying either *β* or *γ* has an approximately equal effect. Thus, the product *βγ* is the more relevant optimization parameter in this intermediate region of the *β–γ* space.

Overall, there are three distinct regions in the *β–γ* parameter space: i) region with high *β* and modest *γ* where *τ* is insensitive to *β*, ii) region with high *γ* and modest *β* where *τ* is insensitive to *γ*, and iii) region with intermediate *β* and *γ* values where *τ* is equally sensitive to *β* and *γ*. Region 1 is avidity-controlled, Region 2 is consumption-controlled, and Region 3 has dual sensitivity. As seen in Figure 5B, the TfR and the LDLR are in the avidity-controlled region, whereas the VtgR is in the consumption-controlled region. In contrast, the EGFR occupies the dual sensitivity region of the parameter space. Thus, avidity modulates the behavior of the TfR and LDLR systems, whereas the consumption coefficient controls the function of the VtgR. As we show in the Discussion section, for dual-sensitivity receptors such as the EGFR, endocytic downregulation is an optimal strategy to improve response accuracy.

## Discussion

Understanding how cells adjust their internal molecular concentrations and reaction rates to accomplish specific tasks is one of the important goals in the emerging field of systems biology [59,60]. Given the ubiquity of receptor-mediated signaling and transport, the identification of design principles for receptor systems is likely to be of significant value for understanding and learning how to modify the mechanisms by which cells process information. The problem we are addressing is analogous to the identification of structure*–*function relationships in proteins. In the current scenario, the reaction network and the parameters correspond to the “structure” of the receptor system, while the manner in which the receptor system responds to ligand inputs corresponds to its “function.” By establishing such “structure*–*function” relationships for biomolecular networks, it should eventually be possible to determine the function of the network simply by examining its “structure” and vice versa.

In this study, we employed a generalized mathematical model to comparatively explore the design principles of signal transduction and transport receptors. Our simulations revealed that the relationships between the individual kinetic parameters and the dynamics of cell surface receptors are complex. In particular, the function of the EGFR was found to be very sensitive to the extent of endocytic downregulation with the information processing accuracy of this system increasing with the *k_e_*/*k_t_* ratio. Although the receptor systems considered here displayed unique patterns of sensitivity to individual parameters, these relationships can be placed in a unified framework by considering the dimensionless equations governing the model. This generalized treatment reveals that the efficiency and robustness of cell surface receptors depend upon two distinct dimensionless parameters: the specific avidity *γ* and the consumption coefficient *β*. Based on their dependence on these parameters, we find that receptor systems can be classified as being i) avidity-controlled, ii) consumption-controlled, or iii) dual-sensitivity systems. The TfR and the LDLR receptor systems are avidity-controlled. The VtgR system is consumption-controlled. Finally, the EGFR is a dual-sensitivity receptor.

An extremely informative approach for analyzing receptor systems was to use relaxation time *τ* as a measure of their “efficiency.” This allowed us to map the relative efficiency of receptors in parameter space to understand some of their design principles (see Figure 6). In Figure 6, *β–γ* parameter pairs that give rise to equal values of *τ* are connected by contour lines. The region with the smallest *τ* values (i.e., fastest response time) is the region where both *β* and *γ* are high (Figure 6, upper right-hand corner). The shapes of the contour lines in the plot indicate the local direction in which a receptor system would have to move to decrease its relaxation time; the best strategy being to move in a direction perpendicular to the local contour lines. The avidity-controlled, consumption-controlled, and dual-sensitivity regions have been demarcated in the plot using contours for the relative sensitivity *r_s_* drawn at the *r_s_* values of 2 (boundary between avidity-controlled and dual-sensitivity) and 0.5 (boundary between dual-sensitivity and consumption-controlled). We were also able to use this approach to map the regions in the parameter space with the highest overall robustness (overall sensitivity *m_s_* < 0.8) and sensitivity (*m_s_* > 1.2) computed as described in the Methods section.

**Figure 6:**
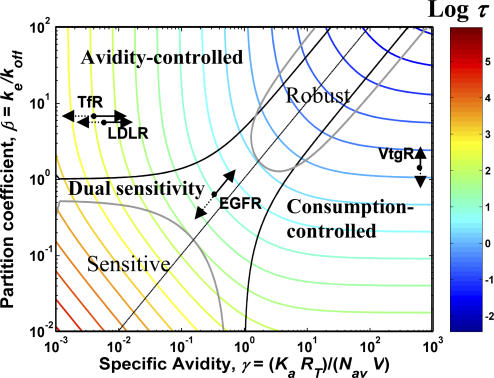
Control Parameters and Their Effect on the Response of Receptor Systems The parameter values for EGFR, TfR, LDLR, and VtgR are shown overlaid on a contour plot of the relaxation time. Smaller relaxation times correspond to larger system efficiencies. Arrows depict the effect of changing the respective control parameters on the response for each of the receptor systems. The control parameters are: i) the specific avidity, *γ*, for TfR and LDLR; ii) the consumption, *β*, for VtgR; and iii) the downregulation—*D* with *β* and *γ* being altered simultaneously for the EGFR. The most efficient manner in which the response of a given receptor system can be improved is by traveling in a direction normal to the contour lines. Figure 6 illustrates how each of the control parameters can be identified based on their ability to move the system in the appropriate direction in the parameter space. The length of the arrows provides a sense as to which receptors are the most sensitive to parameter changes. The sensitivity is in the order: TfR > LDLR > EGFR > VtgR. Solid arrows indicate the effect of increasing the control parameter, while the broken arrows show the effect of decreasing the parameter. The contours demarcating the most robust (*m_s_* < 0.8) and the most sensitive (*m_s_* > 1.2) regions of the parameter space are indicated in gray.

In this study, we computed the relaxation time based on the response of receptor systems to a ligand impulse of magnitude 0.01*K_D_*. However, we have also performed numerical integration of our ODE system to obtain the relaxation time at a ligand dose of *K_D_* nM. The parameter dependency of the relaxation time seen at this higher ligand dosage is qualitatively similar to that reported in Figures 5A and 6 (unpublished data). Thus, our conclusions would still hold true even at the higher ligand concentrations.

As seen in Table 1, the TfR has a small *γ* value and a large *β* value. This places it in the avidity-controlled regime (Figure 6). In retrospect, as *γ* is inversely proportional to *V*, this explains why the TfR was sensitive to the volume and not to *k_e_* (Figures 3B and 4B). We note that increasing the specific avidity *γ* improves the response of the TfR and moves it in the direction toward the global efficiency maximum (Figure 6). The specific avidity is by definition given as *γ* = *K_a_R_T_*/(*N_av_V*). Because *γ* is proportional to *R_T_*, an effective regulatory strategy to improve uptake accuracy and to achieve robust ligand uptake would be to modulate the receptor expression level at the plasma membrane, *R_T_*. Interestingly, several experimental observations support the existence of such a strategy: the expression level of the TfR is modulated at the transcriptional level [31,32], and the number of cell surface TfR molecules are rapidly modulated by membrane translocation following growth factor treatment [34,35]. The LDLR system also occupies the avidity-controlled region of the parameter space with the TfR system (Figure 6). Hence, the impulse responses of these receptor systems display identical parameter dependencies (Figures 3 and 4). The LDLR also displays a similar regulation pattern to the TfR in that its expression level is transcriptionally modulated following the addition of hormones and growth factors [39–42].

An important reason that the TfR and the LDLR are in the avidity-limited region of the *β–γ* parameter space is that the low ligand off-rate of these receptors gives them a relatively large *β* value. Receptor*–*ligand binding parameters are strongly influenced by ligand size and structure. Growth factors are small proteins and thus tend to have a high association rate with their receptors. In addition, the small ligand size leads to a smaller receptor*–*ligand contact area and may contribute to the relatively high receptor*–*ligand off-rates generally seen for these molecules. On the other hand, transport receptors engage molecules that are usually much larger than growth factors. Their association rates are likely to be smaller due to both steric constraints and lower diffusion coefficients. Conversely, these molecules tend to have smaller dissociation rates by virtue of their ability to establish multiple contacts with their receptors. Low receptor off-rates are seen for a number of transport molecules, such as transferrin [61], low-density lipoprotein [14], and asialooromucocoid [62].

Unlike the case with other transport receptors, the VtgR has a very high *γ* value and an intermediate *β* value (Table 1). This places it in the consumption-limited regime where control of the response is primarily a function of *β* (Figure 6). Hence, altering the *γ* value for this receptor system would have a negligible effect on its performance. This is in agreement with the volume sensitivity results for this receptor (Figure 4D). The high *γ* value is primarily caused by the high densities of VtgR found at the surface of oocytes, to which these receptors are typically restricted. High receptor densities are likely an evolutionary consequence of the need to maximize vitellogenin uptake despite the limited surface-to-volume ratio of oocytes. Nevertheless, increasing *β* improves the response of the VtgR and moves it in the direction toward the global efficiency maximum (Figure 6). Because *β = k_e_/k_off_*, the uptake efficiency of the VtgR can ideally be improved by increasing *k_e_* given that *k_off_* is already negligible [47]. In agreement with this notion, hormonal regulation of the VtgR system has been found to be at the level of modulating *k_e_* [48].

The *β* and *γ* values of the EGFR place it in the dual sensitivity region—a region of the parameter space where the receptor system is equally sensitive to changes in *β* and *γ* (Figure 6). This finding explains the results seen in Figures 3A and 4A for the EGFR wherein both *k_e_* and volume were found to have a modulating influence on the impulse response. Given its dual sensitivity, in a local sense, increasing the product *βγ* would lead to an improvement in the EGFR response. However, the best strategy for moving the system to the global efficiency maximum would be to move up the diagonal shown in Figure 6. This can be accomplished by simultaneously increasing both *β* and *γ* while keeping the ratio *β/γ* a constant. However, *β = k_e_/k_off_*, and it can only be increased by reducing ligand dissociation rates or increasing receptor internalization rates. The former parameter is constrained by both the small size of growth factors and the free energy needed for inducing receptor conformational changes [63]. On the other hand, if the internalization rate of both occupied and empty receptors were the same (i.e., *k_e_* = *k_t_*), then increasing *k_e_* would have the effect of decreasing *γ* because *R_T_* = *Q_R_*/*k_t_*. This strategy works for the VtgR because it is in the consumption-controlled region, but would not be effective in the case of the EGFR. Indeed, the only effective strategy for a dual-sensitivity receptor is to uncouple the internalization rate of the occupied receptor *k_e_* from the internalization rate of empty receptors *k_t_*. This provides the means by which to increase *β* without a concomitant decrease in *γ*. The diversity of regulatory mechanisms that have evolved to uncouple *k_e_* from *k_t_* attests to the strong selective advantage of this design strategy for signaling receptors.

The response characteristics of the EGFR system can be improved by either increasing *k_e_* or decreasing *k_t_* or both. The local symmetry of the dual-sensitivity region implies that each of these strategies would have the same benefit when it comes to improving response characteristics. However, when we consider order-of-magnitude changes in the system parameters, the ideal strategy would be to move along the diagonal, which can be accomplished by increasing *k_e_/k_t_* while maintaining the constraint *k_e_k_t_* = constant (i.e., *β/γ* = constant). This amounts to increasing downregulation by *simultaneously* increasing *k_e_* and decreasing *k_t_*. This suggests that signaling receptors should greatly benefit from decoupling the internalization of receptor*–*ligand complexes from the internalization of free receptors. Indeed, in agreement with this concept, it has been shown that the internalization of empty EGFR can be regulated independently of occupied receptors [64].

Traditionally, endocytic downregulation has been proposed to be a mechanism to prevent cells from “overresponding” to a growth factor stimulus, with a perceived role analogous to a pressure relief–valve system. Furthermore, the term “downregulation” implies that once the receptor system is induced to respond to ligand, the system enters a refractory period wherein further stimulation would not result in a biological outcome. However, it is very unlikely that cells would ever see a dose of ligand sufficient to downregulate a significant number of surface receptors to transform the cells to a refractory state. For example, quantitative analysis of autocrine systems has shown that cells produce sufficient ligand to occupy only a small fraction of available receptors [17]. Instead of seeing endocytic downregulation as a relief valve, our analysis suggests that it is more analogous to a negative feedback control module that improves the response accuracy of the system. Endocytosis itself is clearly a mechanism to ensure that ligand molecules that enter the extracellular space are promptly cleared. However, the differential endocytosis of occupied versus empty receptors appears to be primarily a regulatory mechanism to enhance the temporal fidelity of signaling receptors.

The eventual output of a signaling system is a cellular response such as cell proliferation or migration. It is possible that different cellular responses may display varying degrees of coupling with the temporal ligand input. Based on our analysis, we propose that the upstream portion of a signaling receptor system such as the EGFR is designed for accurate synchronous signal processing so that a change in ligand concentration is accurately reflected in the number of surface complexes. Downstream cellular responses that are intimately coupled to the number of surface complexes would also display a match with the input signal. Responses such as cell migration could in fact follow this strategy where transient inputs result in transient outputs. On the other hand, the number of internalized complexes reflects an integration of the input signal from the time of endocytosis to the time of receptor inactivation [65]. If the eventual cellular response were to be coupled to the number of internalized complexes, then the cellular response would be an integration of the input signal.

Assessing the design principles of receptor systems requires establishing quantitative measures of receptor function. Drawing upon analogies with engineered systems, we can employ two qualitatively different measures to evaluate a system: efficiency and robustness. Whereas it is intuitively evident that the efficiency is a desirable system trait, the situation with respect to robustness is less certain. For example, we found that none of the receptor systems we investigated were located in the maximally robust region of the parameter space (Figure 6). However, we note that a receptor system that is completely robust to all parameter variations cannot be effectively regulated. For example, hormonal modulation of receptor expression levels and internalization rates would have no significant effect on system response. Hence, maximal robustness might not necessarily be a desirable outcome during receptor evolution. There are situations, however, where robustness to a specific parameter, such as volume changes, would be a desirable trait. For example, the EGFR system operates in a wide variety of physical contexts both during development and in the adult organism. Robustness to volume changes would allow this system to function with comparable efficiencies in numerous circumstances. We note that there is experimental evidence to support the fact that the extracellular volume can affect EGFR-mediated signaling. It has been shown previously that interstitial volume changes due to mechanical stress can result in a proportional change in the EGFR-mediated ERK phosphorylation levels [66]. It is possible that the interstitial volume in these experiments is greater than 4 × 10^−11^ liters/cell, which would place the EGFR system in the volume-sensitive portion of the parameter space (see Figure 4A). Alternately, low levels of volume sensitivity coupled with the amplification provided by the MAPK cascade could result in the proportional coupling between ERK activation and volume change seen in the experiments. In summary, there is clearly a tradeoff between robustness and regulation. Understanding which specific receptor characteristics are robust could provide important insights into the evolutionary pressures on a particular receptor system.

The inherent tradeoff between efficiency and robustness can be used to further categorize the control strategies used by our specific receptor systems. We suggest that the TfR and the LDLR as seen in Figure 6 are in a basal state of operation where the efficiency (response speed) is low. However, this comes with the advantage that these systems can be easily regulated at the level of receptor expression with a change in receptor expression leading to a substantial relative change in system efficiency. Thus, hormones and growth factors can switch these systems to an “on state” where the efficiencies are higher. Thus, it is possible to view the TfR and LDLR as receptor-controlled systems where the response control is at the level of the receptor. On the other hand, the EGFR system as seen in Figure 6 inherently has a higher efficiency and a lower sensitivity to parameter changes. Further, to the best of our knowledge we are not aware of instances where the EGFR system is acutely regulated analogous to the hormonal regulation seen in the TfR and LDLR systems. Thus, the EGFR system by virtue of its inherently higher information processing accuracy is a ligand-controlled system where the system response is tightly coupled to variations in ligand concentration.

## Materials and Methods

### Construction of the mathematical model.

Figure 1 presents a schematic description of the reaction system analyzed in this paper. The governing equations corresponding to this reaction system are presented in Equation 1 (Results section). We made several simplifying assumptions in constructing our mathematical model. First, we restricted our analysis to the formation of cell-surface receptor*–*ligand complexes and used the number of surface complexes as the system readout. It has been shown that in the case of the EGFR and other signaling receptors, the biological response is proportional to the number of receptor*–*ligand complexes at the cell surface, and thus they constitute a good readout for cell responses. This does not imply that the surface receptors are the source of the signal. It only means that since the formation of surface complexes is a precursor to subsequent downstream events, it can be used to assess the magnitude of signal transduction. A second simplification in our analysis is that we restrict our model to internalization and do not explicitly include subsequent receptor/ligand trafficking phenomena such as recycling. For the transport receptors, recycling increases the capacity of the ligand internalization system and can be approximated as an increase in apparent avidity. For signaling receptors, receptor recycling does not appear to play a major role in modulating the efficiency of information processing [67]. Restricting our model to internalization alone reduced the dimensionality of our parameter space and simplified the comparison of the different receptor systems.

### Numerical solution of the governing equations.

The governing equations (Equation 1) were integrated using the MATLAB (The Mathworks, http://mathworks.com) stiff equation solver ode15s to obtain the response to various inputs such as impulses and square waves. The parameter values used for the various receptor systems are listed in Table 1. In simulations examining the contribution of a single parameter to the receptor dynamics, the chosen parameter was varied in a specified range while all other parameters were held fixed at the values listed in Table 1. For impulse response simulations where the response to an impulse of magnitude 0.01*K_D_* was desired, *f*(*t*) was set equal to zero for the entire simulation, and the initial conditions used were *R* = *R_T_*, *C* = 0, and *L* = 0.01*K_D_*. The dimensionless response was computed as C^*^ = *C*/*R_T_* and plotted as a function of time. The repeating unit of a time-periodic square wave is an activation phase where ligand enters the system at a constant rate followed by a relaxation phase where the ligand entry rate is zero. For square wave inputs, the *f*(*t*) value for the activation phase was chosen such that the free receptor number *R* would drop to a steady state value of 0.95*R_T_* if the activation phase was sustained. By setting the rates of change of the various species in Equation 1 to zero, we can show that this condition yields the expression, *f*(*t*) = 0.05*Q*_R_/(*N_av_V*) M/min for the activation phase. In the relaxation phase of the square wave, *f*(*t*) was set equal to zero. For the square-wave simulations, the response was normalized based on the maximum value of *C* reached in the simulation window, as C_n_^*^ = *C/C_max_* to facilitate comparison of the time lags between the input and the output for various parameter values*.* Square waves are characterized by three parameters, namely the magnitude (*f*(*t*) in the activation phase) and the duration of the activation and the relaxation phases. To generate noisy square-wave inputs, all three of these parameters were chosen from a standard normal distribution with a 50% standard deviation.

Simulations where the ligand uptake dynamics of the various receptor systems were compared were performed using a ligand impulse of magnitude 0.01*K_D_*. In these simulations, the concentration of internalized ligand based on the extracellular volume was computed using the equation d*L_i_/*dt = (*k_e_C*)/(*N_av_V*). The internalized ligand concentration was expressed as a percentage of the total externally added ligand as percent internalized ligand = [*L_i_*/(0.01*K_D_*)] × 100.

### Computation of the relaxation time and its sensitivity to *β* and *γ*.

The relaxation time *τ* is defined as the time taken for the impulse response to decay to a value 1/e = 1/2.7183 of the maximum. To compute the *τ* value, we need to first compute the impulse response C^*^(t^*^), which can be done by numerical integration of the dimensionless model equations (Equation 2 in the Results section). Subsequently, the *τ* value can be obtained by interpolation so as to satisfy the relationship C^*^(*τ*) = C^*^_max_/e, where C^*^_max_ is the maximum value reached by the response. Because the impulses employed here are quite small (magnitude = 0.01), the response C^*^(t^*^) can be reasonably approximated by obtaining an analytical solution for the system of equations obtained by linearization of Equation 2 around the initial steady state in the absence of ligand, namely R^*^ =1, C^*^ = 0, L^*^ =0. This procedure leads to two linear ordinary differential equations for the rates of change of C^*^ and L^*^, respectively, each of which are independent of R^*^. These equations are: dC^*^/dt^*^ = − (1 + *β*)C^*^ + L^*^; dL^*^/dt^*^ = γC^*^ − γL^*^ + f^*^(t^*^). Because *β* and *γ* are greater than zero, eigenvalues for this equation system are always real, distinct, and negative. It can thus be shown that the solution for C^*^ involves a double exponential and is given by:

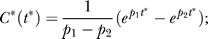
where

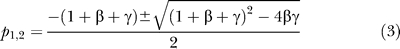
Equation 3 clearly demonstrates that the response of the system is only a function of the *β* and *γ* parameters. The primary advantage of obtaining an analytical solution is the substantial gain in computation speed compared with the numerical solution. The time resolution for generating the response curve C^*^(t^*^) needs to be reasonably small so that interpolation to obtain the *τ* value yields accurate results. Our tests showed that, for an impulse of magnitude 0.01, the analytical solution yielded relaxation times that were within 4% of the results obtained using the numerical solution over the entire range of *β* and *γ* values (unpublished data). We therefore computed the relaxation time *τ* for a range of *β* and *γ* values by using Equation 3 for C^*^(t^*^) and then interpolating the results to obtain *τ*.

We also computed the sensitivity of the relaxation time to changes in the system parameters *β* and *γ* by numerically evaluating the derivatives ∂*_β_τ* = (*β*/*τ*)∂*τ*/∂*β* = ∂ln*τ*/∂ln*β* and ∂*_γ_τ* = (*γ*/*τ*)∂*τ*/∂*γ* = ∂ln*τ*/∂ln*γ*. The sensitivity indices ∂*_β_τ* and ∂*_γ_τ* quantify the percentage change in *τ* for a 1% change in *β* and *γ*, respectively, and can thus be characterized as specific sensitivities. For computing these sensitivity indices, we generated a linear grid for the variables (*b*, *g*). Logarithmic *β* and *γ* values at a particular (*b_i_*, *g_j_*) grid point are given by *β_i_* = log_10_(*b_i_*) and *γ_j_* = log_10_(*g_j_*). The *i* and *j* indices vary from 1 to 100; that is, the size of the grid was 100 × 100. The linear grid spanned the values from −2 to 2 and −3 to 3 for the *b* and *g* variables, respectively. Thus, in our computations, *β* and *γ* values were varied in the range 10^−2^ to 10^2^ and 10^−3^ to 10^3^, respectively, on a logarithmic scale. These ranges were chosen based on our analysis of experimental data reported in the literature for receptor–ligand binding and trafficking. We computed the numerical derivatives ∂*τ*/∂*b*|_(*i*, *j*)_ and ∂*τ*/∂*g*|_(*i*, *j*)_ at each grid point (*b_i_*, *g_j_*). The relative sensitivities to *β* and *γ* were then computed using these values as ∂*_β_τ*|_(*i*, *j*)_ = [*β_i_/τ*(*i*, *j*)] ∂*τ*/∂*b*|_(*i*,*j*)_/ln10 and ∂*_γ_τ*|_(*i*, *j*)_ = [*γ_j_/τ*(*i*, *j*)] ∂*τ*/∂*g*|_(*i*, *j*)_/ln10. Once the relative sensitivities were computed, we determined the ratio of the sensitivities *r_s_*(*i*, *j*) = [∂*_γ_τ*|_(*i*, *j*)_]/ [∂*_β_τ*|_(*i*, *j*)_]. The *r_s_*(*i*, *j*) values indicate the relative sensitivity of the relaxation time to changes in *γ* and *β*. Small *r_s_* values would indicate that the relaxation time is more sensitive to changes in *β*, and large values would indicate greater sensitivity to *γ*. Finally, we computed the overall magnitude of the sensitivity of the relaxation time to changes in *β* and *γ* as *m_s_*(*i*, *j*) = [(∂*_β_τ*|_(*i*, *j*)_)^2^ + (∂*_γ_τ*|_(*i*, *j*)_)^2^]^1/2^. *m_s_*(*i*, *j*) quantifies the robustness of the system in the face of cumulative parameter variations. Small values indicate robustness, and large values are a sign of increased sensitivity to parameter changes.

## Supporting Information

### Accession Numbers

Accession numbers used in this paper from UniProt (http://www.uniprot.org) are for: EGFR, Homo sapiens (Q504U8), TfR, H. sapiens (P02786), LDLR, H. sapiens (Q9UH51), and VtgR, Xenopus laeviss (Q42126) and (Q6NS01).

## Abbreviations

EGFR: epidermal growth factor receptor
LDLR: low-density lipoprotein receptor
TfR: transferrin receptor
VtgR: vitellogenin receptor
